# PTH Derivative promotes wound healing via synergistic multicellular stimulating and exosomal activities

**DOI:** 10.1186/s12964-020-00541-w

**Published:** 2020-03-09

**Authors:** Yi-Fan Shen, Jing-Huan Huang, Kai-Yang Wang, Jin Zheng, Lin Cai, Hong Gao, Xiao-Lin Li, Jing-Feng Li

**Affiliations:** 1 Department of Orthopaedic Surgery, Shanghai Jiao Tong University Affiliated Sixth People’s Hospital, Shanghai, People’s Republic of China; 2 Department of Orthopedics, Zhongnan Hospital of Wuhan University, Wuhan, People’s Republic of China; 3 Department of Neurology, Union Hospital, Tongji Medical College, Huazhong University of Science and Technology, Wuhan, People’s Republic of China

**Keywords:** PTH, Multifunctional factor, Diabetic wound, Exosomes, Synergistic effect

## Abstract

**Background:**

Diabetic wounds are a disturbing and rapidly growing clinical problem. A novel peptide, parathyroid hormone related peptide (PTHrP-2), is assumed as multifunctional factor in angiogenesis, fibrogenesis and re-epithelization. This study aims to test PTHrP-2 efficiency and mechanism in wound healing.

**Methods:**

Through repair phenomenon in vivo some problems were detected, and further research on their mechanisms was made. In vivo therapeutic effects of PTHrP-2 were determined by HE, Masson, microfil and immunohistochemical staining. In vitro direct effects of PTHrP-2 were determined by proliferation, migration, Vascular Endothelial Grown Factor and collagen I secretion of cells and Akt/ Erk1/2 pathway change. In vitro indirect effects of PTHrP-2 was study via exosomes. Exosomes from PTHrP-2 untreated and treated HUVECs and HFF-1 cells were insolated and identified. Exosomes were co-cultured with original cells, HUVECs or HFF-1 cells, and epithelial cells. Proliferation and migration and pathway change were observed. PTHrP-2-HUVEC-Exos were added into in vivo wound to testify its hub role in PTHrP-2 indirect effects in wound healing.

**Results:**

In vivo, PTHrP-2 exerted multifunctional pro-angiogenesis, pro-firbogenesis and re-epithelization effects. In vitro, PTHrP-2 promoted proliferation and migration of endothelial and fibroblast cells, but had no effect on epithelial cells. Therefore, we tested PTHrP-2 indirect effects via exosomes. PTHrP-2 intensified intercellular communication between endothelial cells and fibroblasts and initiated endothelial-epithelial intercellular communication. PTHrP-2-HUVEC-Exos played a hub role in PTHrP-2 indirect effects in wound healing.

**Conclusion:**

These findings of this study indicated that PTHrP-2, a multifunctional factor, could promote wound healing via synergistic multicellular stimulating and exosomal activities.

**Graphical abstract:**

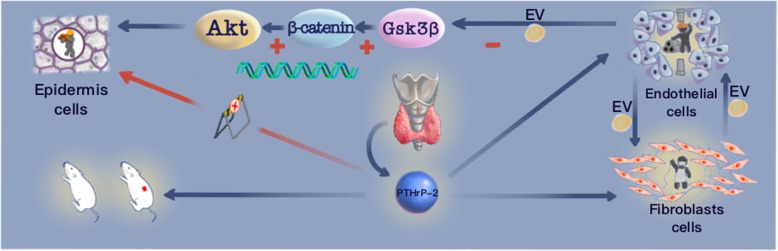

Video abstract.

## Background

With the improvement of people’s living standards and the acceleration of urbanization, people’s diet and lifestyle have changed, leading to an increasing incidence of diabetes worldwide [1]. Diabetic wound is one of the most common complications of diabetes which costs billions of dollars, and causes a high socioeconomic burden [2]. Traditional diabetic wound therapies include dressing changes, repeated debridement and amputation [3]. Regenerative approaches, such as approaches using cytokines and hydrogels for tissue engineering are being developed and hold promise for wound healing [4, 5]. Currently, cytokines such as epithelial cell growth factor (EGF) and basic fibroblast growth factor (b-FGF) are widely used in clinic and promote healing [6, 7]. However, these cytokines are often expensive and have strict limitations regarding use currently used factors exert their healing abilities in either the dermal layer or corium layer of wounds [8].

Skin tissue contains two layers, the dermal layer and corium layer [9], and is composed of three major cell types: epithelial cells, fibrocytes and endothelial cells [10–12]. Fibrocytes, fibers, endothelial cells and capillaries construct the corium layer, whereas epithelial cells compose the dermal layer. Three cell types are closely related in anatomic and functional aspects. Collagens form the major scaffold for the corium layer are important in wound healing [11]. Endothelial cells and epithelial cells attach within or on the scaffold and form capillaries and the dermal layer. The capillaries supply oxygen and nutrients to the fibrocytes and epithelial cells, whereas the dermal layer protects the corium layer from bacteria and radiation [13]. These three cell types communicate and interact with one another via paracrine actions [14]. Therefore, an optimal approach might involve the target of all three cell types to promote wound healing via synergistic effects.

PTH, a hormone secreted by the parathyroid, is an important factor in bone turnover and calcium phosphorus metabolism [15, 16]. It is used as an anabolic agent in osteoporosis therapy. Previous studies by other scholars and our research group have shown that PTH can not only stimulate osteoblasts but also endothelial cells and promote angiogenesis [17]. In addition, there is evidence that PTH promotes fibrogenesis and collagen deposition [18, 19]. PTH1R, a major receptor of PTH, has been found to be expressed in endothelial cells, fibroblasts and epithelial cells, which indicate the activating basis of application of PTH in skin tissue [20–23]. Therefore, we hypothesize that PTH might promote wound healing via multicellular stimulation and multilayer skin tissue repair.

In present study, we tested this hypothesis. We developed a novel PTH derivative, PTHrP-2 [24], and applied it to diabetic wounds in rats. Macroscopic, histological and radiological analyses verified that PTHrP-2 can significantly promote wound healing via proangiogenesis, profibrogenesis and re-epithelization. In vitro results confirmed these findings. Endothelial cells and fibroblasts were activated by PTHrP-2 and showed enhanced proliferation, migration and angiogenic or fibrogenic activity. However, epithelial cells did not show any activation under PTHrP-2 treatment. The difference between in vivo results and in vitro results led use to hypothesize that PTHrP-2 promotes re-epithelization via indirect intercellular communications.

The exosome is a recently discovered extracellular vesicle that is important in intercellular communications [25]. Recent studies have reported that drugs, hormones and environmental factors could alter exosome contents and intercellular communication [26]. Therefore, we attributed in vivo re-epithelization promotion by PTHrP-2 to exosome-mediated changes in intercellular communication. Exosomes were isolated from PTHrP-2-stimulated endothelial cells. Self-activation and fibroblast- and epithelial cell-activation by exosomes from PTHrP-2-stimulated endothelial cells were observed in vitro and in vivo studies.

## Materials and methods

### Synthesis and loading of PTHrP-2

To synthesize PTHrP-2, the FMOC/tBu solid-phase method was used [27]. The whole sequence of PTHrP-2 is S [PO4] VSEI-QLMHN-LGKHL-NSMER-VEWLR-.

KKLQD-VHNF-EEE. The three glutamic acid (Glu) residues at the C terminal and the phosphorylated serine (Ser) residue at the N-terminal are the major variations in PTHrP-2 relative to the 1–34 amino acids of PTH. Gel filtration was applied for initial purification of the crude peptide. In order to release PTHrP-2 sustainably in vivo, PTHrP-2 loading was applied in calcium alginate hydrogel. According to previous studies [28, 29], 2% sodium alginate solution was mixed with PTHrP-2, and 1.5% CaCl2 solution was mixed into gel. The final product was PTHrP-2@Ca-Alg.

### In vivo study

#### Induction of diabetes and excisional wound splinting model preparation

Forty-five male Sprague-Dawley rats aged 8 weeks were selected to construct a diabetic rat model according to a previously established method. All protocols obtained approval from the Animal Care and Experimental Committee of the Shanghai Jiao Tong University Affiliated Sixth People’s Hospital. Before operation, the rats fasted for one night to measure the baseline blood glucose levels. Streptozotocin (65 mg/kg b.w., i.p.) was intraperitoneally injected, and blood glucose levels were measured at three time points, on days 1, 3 and 7. After observation for 2 weeks, 24 rats with glucose levels of over 300 mg/dl were selected as experimental rats for follow-up operation. According to the results of the in vitro experiment, the diabetic rats were divided into three groups, control, Ca-Alg, and PTHrP-2@ Ca-Alg, to evaluate the ability of PTHrP-2@ Ca-Alg to repair diabetic skin wounds.

#### Animals and surgical procedure

A rodent model of full-thickness skin wounds in diabetes was established. After the wound was successfully established, Ca-Alg and PTHrP-2@ Ca-Alg were placed on the wound surfaces of the animals. After surgery, sterile gauze was used to fix the wound surface. The rats were observed every day to ensure that the dressings were intact. After the operations, the animals were placed in a controlled temperature environment and continued to be fed with the diabetic diet, and their bedding was replaced every day.

#### Measurement of wound size reduction

Postoperative photos were taken with a camera (Canon, Japan) at the following four time points: day 0, day 3, day 7 and day 14. A model diagram of wound repair was constructed, and the changes in wound area and repair status were analyzed by ImageJ. The amount of wound closure was determined using the formula percent wound size reduction = ¼ [(A0-At)/A0] 100, where A0 was the initial wound area (t ¼ 0), and At was the wound area at each time point.

#### Microfil perfusion and microcomputed tomography

Microfil was used to evaluate neovascularization during wound healing in the diabetic rats. The experimental rats were euthanized 14 days after surgery. The hair was removed from the chest of each rat, and scissors were used to cut open the chest. After clamping the descending aorta and incising the inferior vena cava, the left ventricle was penetrated with an angiocatheter. Then, 100 ml of heparinized saline and 20 ml of Microfil (MV-122; Flow Tech, USA) were successively perfused at 2 ml/min. To ensure the polymerization and solidification of the contrast agent, the experimental samples were incubated at 4 °C overnight. On the second day after the operation, the sample was pruned and scanned with microcomputed tomography (Micro CT) at a resolution of 9 mm to detect new blood vessels. Using 3D Creator software, 3D images were reconstructed. The blood vessel area and number of blood vessels in the wound were also determined using this software.

#### Histologic, immunohistochemical and immunofluorescence analysis

For histology, the samples were dehydrated, embedded in paraffin and sliced into sections (~ 6 μm thick). Neuroepithelial length and collagen deposition were observed via hematoxylin and eosin (H&E) and Masson’s trichrome staining. Immunohistochemistry and immunofluorescence were applied to observe angiogenesis and fibroblasts in the wound field. For immunohistochemistry, the sections were rehydrated and treated with antigen retrieval. After incubation with the primary antibody against CD31 (1:200, Abcam, Cambridge, UK) at 4 °C overnight, the sections were incubated with a biotinylated secondary antibody and an ABC complex and stained with DAB substrate. All sections were counterstained with hematoxylin and observed under a light microscope. For immunofluorescence, the sections were rehydrated and blocked with 1.5% goat serum (Merck-Millipore). After incubation with primary antibodies against CD31 (1:200, Abcam, Cambridge, UK) and α-SAM (1,50, Abcam, Cambridge, UK) at 4 °C overnight, the sections were incubated with Alexa Fluor 488- and Cy3-conjugated secondary antibodies and DAPI (Sigma-Aldrich) for visualization. The sections were observed via confocal laser scanning microscopy. Angiogenesis was determined in six sections from different samples. For each section, six high-power fields containing the entire portion of the wounds were randomly observed, and the newly formed blood vessels were evaluated. All counting procedures were conducted separately by two pathologists.

### PTHrP-2 effects on endothelial cells, fibroblasts and epithelial cells

#### Cell culture

HUVECs (Sciencell Research Laboratories, San Diego, CA, USA) were cultured in complete endothelial cell medium (ECM, Sciencell, USA) containing 2.5% fetal bovine serum (FBS, Sciencell), 1% endothelial cell growth supplement (ECGS, Sciencell) and 1% penicillin-streptomycin (P/S, Sciencell). Only the HUVECs from early passages (passages 2~7) were used in the subsequent experiments. HFF-1 cells (SCSP-109, Stem Cell Bank, Chinese Academy of Sciences) and human immortalized epidermal cells (HaCaTs) (AD4013, ATCC) were cultured under humidified conditions in serum-free Dulbecco’s modified Eagle’s medium (DMEM; GIBCO; Invitrogen Pty Ltd., Australia) supplemented with 2.5% FBS (Sciencell) and 1% P/S (Sciencell). Cells were cultured in a humidified 37 °C/5% CO2 incubator.

#### Cell proliferation and migration

The proliferation of HUVECs, HFF-1 cells, and HaCaTs was analyzed with the CCK-8 method. HUVECs were cultured in medium under control conditions or with 0.1 nM, 1 nM or 10 nM PTHrP-2 (*n* = 4). The cells were inoculated in 96-well culture plates (Corning, USA) at a density of 2 × 10^3^ cells per well and cultured for 1, 3 and 7 days according to the different conditions of each group. HFF-1 cells and HaCaTs were cultured under the same conditions, but the initial number of cells was 1.5 × 10^3^. Then, 100 μl of culture medium containing 10% CCK-8 was added to each well of the 96-well plate, and the plates were incubated for 2 h. The absorbance value of each sample was immediately measured at 450 nm by a microtiter plate reader (BioTek, Winooski, USA).

The migration of HUVECs, HFF-1 cells and HaCaTs was tested with a transwell assay (3422, Corning, USA). HUVECs, HFF-1 cells and HaCaTs at a density of 2 × 10^4^ cells were inoculated in the upper chamber and cultured in 200 μl of serum-starved medium, whereas the lower chamber contained 500 μl of complete medium. The cells were fixed and stained for 10 min with 0.1% crystal violet after incubation for 24 h. The migrated cells were photographed by microscopy (Olympus IX 70, Tokyo, Japan) and counted by ImageJ.

#### Angiogenic characters

MatrigelTM (BD Bioscience) was thawed in advance in a 4 °C refrigerator overnight. In precooled 24-well plates, 200 μl of Matrigel was added to each well, and the plates were then incubated at 37 °C for 1 h. HUVECs that had been precultured for 48 h in medium under different conditions were digested with trypsin and counted. A total of 1 × 10^5^ pretreated HUVECs were added to the 24-well plates containing Matrigel, and the samples continued to be cultured in the treated medium. The tube-forming ability of HUVECs was observed by microscopy (Olympus IX 70, Tokyo, Japan) after culture in a humidified 37 °C/5% CO2 incubator for 8 h. Statistical analysis of the number of tubes in the microscope (Olympus IX 70, Tokyo, Japan) photos was carried out with Image J.

After culture in medium under the control condition or with 0.1 nM, 1 nM or 10 nM PTHrP-2 (*n* = 4) for 3 days, HUVECs and HFF-1 cells were fixed with 4% paraformaldehyde for 15 min and then washed with PBS three times. Next, the cells were permeabilized with 0.25% Triton X-100 for 15 min and then blocked with 3% bovine serum albumin (BSA) for 1 h. After washing with Phosphate Buffered Saline (PBS), we added anti-VEGF (1:200, ABclonal, China) and incubated with the cells in a 4 °C refrigerator overnight; then, we added the secondary antibodies in darkness. One hour later, the cells were washed with PBS three times for 5 min each time. The cytoskeletons were then stained with 5 g/ml of phalloidin (1:200, Yeasen, China) at room temperature for 45 min. Then, the slides were washed with PBS three times for 5 min each time. After the final wash, the samples were stained by adding 4′,6-diamidino-2-phenylindole (DAPI, 1:200, Solarbio) in PBS for 10 min, followed by imaging. The cells were visualized using a confocal microscope (Leica, Solms, Germany).

The VEGF secretion from HUVECs was detected by an enzyme-linked immunosorbent assay (ELISA). A total of 1 × 10^5^ HUVECs were seeded in medium under the control condition or with 0.1 nM, 1 nM or 10 nM PTHrP-2 (*n* = 4) in 6-well plates. After the cells were cultured for 3 days, the supernatants of the samples were collected, and the contents of VEGF released from the samples were detected in strict accordance with the manufacturer’s instructions using an ELISA kit (NeoBioscience, China).

#### Fibrogenic characters

Immunofluorescence (Anti-Collagen I, Abcam, UK) and enzyme-linked immunosorbent assay (ELISA) (Human Pro-Collagen I alpha 1 DuoSet ELISA, R&D systems, USA) were used to determine the type I collagen in HFF-1 cells.

#### Western blotting to evaluate angiogenic and fibrogenic characteristics

For Western blotting, exosomes or cells were lysed. The lysates were diluted with 5 × loading buffer at a ratio of 1:5 and heated at 95 °C for 5 min. The protein extracts were separated by sodium dodecyl sulfate-polyacrylamide gel electrophoresis (SDS-PAGE) and transferred to polyvinylidene fluoride membranes (Immobilon P, Millipore, Billerica, USA). The membranes were blocked with 5% nonfat milk or BSA for 1 h and then incubated with primary antibodies overnight at 4 °C and with HRP-linked secondary antibodies for 1 h at room temperature. The protein bands were then visualized using an enhanced chemiluminescence (ECL) substrate kit (Merck Millipore, USA).

#### Exosome isolation

The HUVEC and HFF-1 exosomes were isolated by ultracentrifugation. In brief, when HUVEC and HFF-1 cultures reached 80% confluence, the culture medium was removed, and the cells were washed three times with PBS. Then, serum-free medium was used for culturing. PTHrP-2 was added to the medium of the PTHrP-2-treated group at this time. After 48 h of culture, conditioned media were collected and centrifuged at 300×g for 10 min and 2000×g for 15 min to remove dead cells and debris. The supernatants were then filtered via a 0.22-μm filter (Micropore) and centrifuged at 100000×g for 1.5 h twice. Then, the pellets were resuspended in PBS.

#### Exosome characterization

The morphology of exosomes was identified by TEM (JEM-1400, JEOL, Japan). Western blotting analysis was used to verify the exosome markers Alix, TSG101 (Protein Tech, USA) and CD9 (Abcam, USA). DLS was applied to determine the exosome size distribution. Particle concentration, particle size and the video frame of exosomes were analyzed by a Flow NanoAnalyzer (FNA) (NanoFCM, China) and nanoparticle tracking analysis (NTA) (ZetaView PMX 110, Particle Metrix, Meerbusch, Germany). The protein concentration of exosomes was quantitatively detected by a BCA protein assay kit.

#### Exosome internalization

The purified exosomes were labeled with the red fluorescent dye PKH26 (Sigma-Aldrich, Germany) according to the manufacturer’s protocol. Subsequently, PKH26-labeled exosomes were added to the medium and incubated with HUVECs, HFF-1 cells and HaCaTs for 24 h. Afterwards, the cells were fixed and washed with PBS 3 times and then blocked with QuickBlock™ Blocking Buffer for Immunol Staining (Beyotime, China). The cytoskeleton was then exposed to 5 g/ml of phalloidin (1:200, Yeasen) at room temperature for 45 min. Then, the slides were washed with PBS three times for 5 min each time. After the final wash, the samples were stained by adding DAPI (1:200, Solarbio) in PBS for 10 min and then imaged. The cells were visualized using a confocal microscope (Leica, Solms, Germany).

#### Exosome-mediated intercellular communication

Exosomes were extracted from HUVECs and HFF-1 cells in the PTHrP-2-treated and untreated groups using the method described above. Exosomes from the treated groups (PTHrP-2-HUVEC-Exos and PTHrP-2-HFF-1-Exos) and untreated groups (HUVEC-Exos and HFF-1-Exos) were cultured together with HUVECs and HFF-1 cells, and the proliferation, migration and tube formation experiments were performed as described above.

#### Effects of PTHrP-2-treated-exosomes on HaCaTs

The exosomes extracted above were co-cultured with HaCaTs to evaluate the proliferation and migration ability of HaCaTs according to the methods described above. According to the characterization results of HaCaTs, the mechanisms were explored by Western blot.

#### In vivo validation of PTHrP-2-HUVEC-Exos

The effect of PTHrP-2-HUVEC-Exos in vitro was verified by subcutaneous injection in diabetic rat wounds. Histopathological methods were used to analyze the rats 7 days after operation. HE staining, Masson staining, CD31 immunohistochemical staining and CD31/α-SMA dual immunofluorescence staining experiments were carried out according to the methods described above.

### Statistical analysis

All experiments, both in vitro and in vivo, were repeated at least three times. Data were representative of these experiments and were shown as the mean ± standard deviation (SD). The means of multiple groups were compared with one-way analysis of variance (ANOVA). The independent sample test was used to compare means between two groups. Statistical analysis was conducted using GraphPad Prism software, and *P* < 0.05 was considered statistically significant.

## Results

### Evaluation of PTHrP-2@Ca-Alg effects on wound healing in vivo

**Fig. 1:**
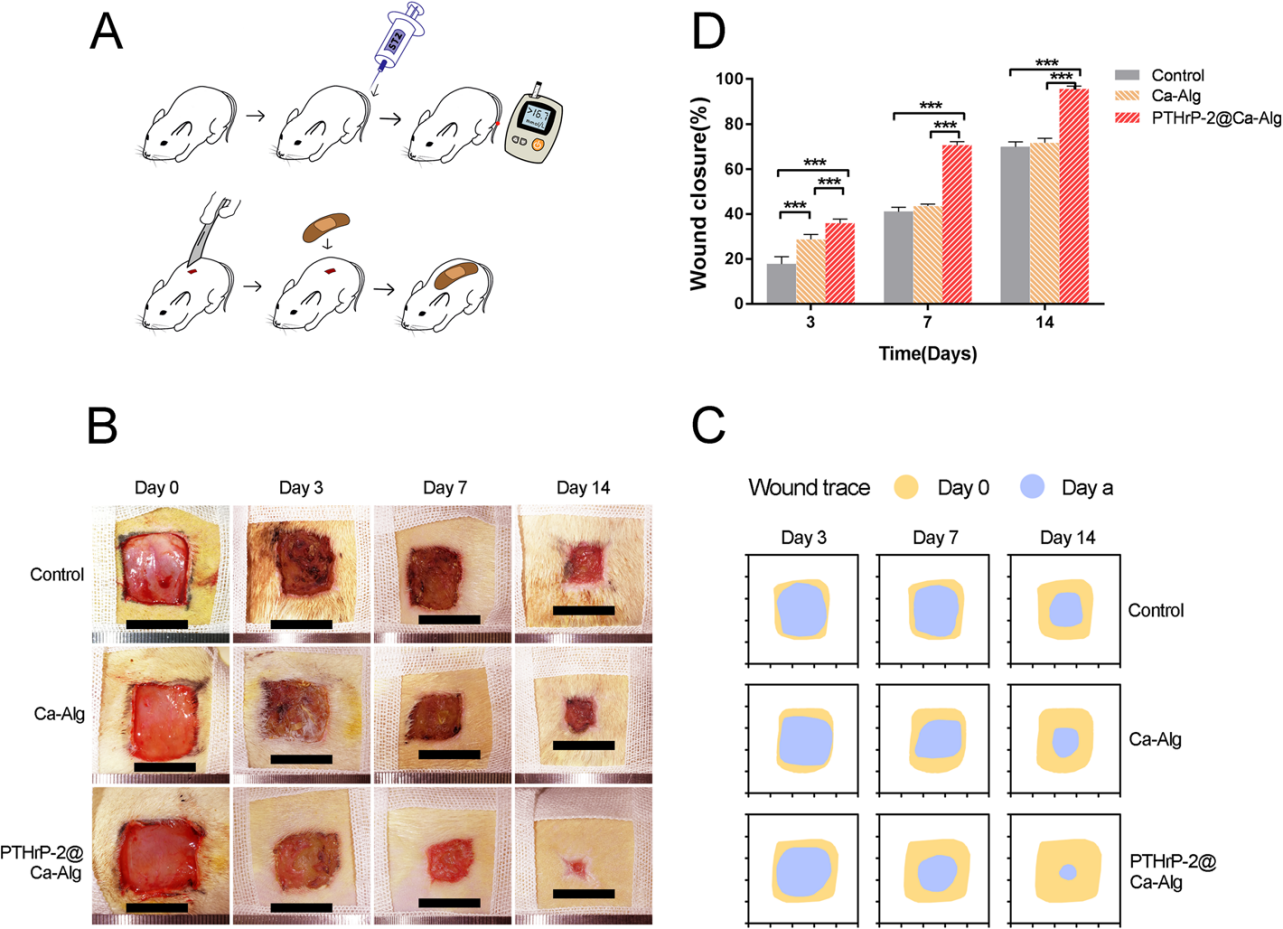
**a** The illustration of the experiment in vitro (**b**) The size change of the wounds made in the dorsal derma of diabetic rat was observed at 0,3,7,14 days after surgery. **c** The change of wound size in different treatment groups. The yellow area is the size of the wound at day 0, and the light blue area is the size of the wound at n (n=, 3,7,14) days. **d** The wound closure rate of each group at 0,3,7,14 days after surgery

Figure 1 a outlines the process of the animal experiment. Figure 1 b showed untreated wounds and wounds treated with Ca-Alg or PTHrP-2@Ca-Alg at 4 time points. Over time, the size of the wounds in all three groups decreased by various degrees, with the wounds in the PTHrP-2 group and the PTHrP-2@Ca-Alg group becoming smaller than those in the untreated group (Fig. 1 c). The wounds treated with PTHrP-2@Ca-Alg were nearly healed by day 14. According to the quantitative data analysis of wound closure (equation [1]), the areas of wounds in the group treated with PTHrP-2@Ca-Alg were smaller than those in the other groups at three time points, and among the three groups, the PTHrP-2@Ca-Alg group exhibited the highest wound healing rate. The process of wound healing in rats was illustrated in Fig. 1d.

**Fig. 2:**
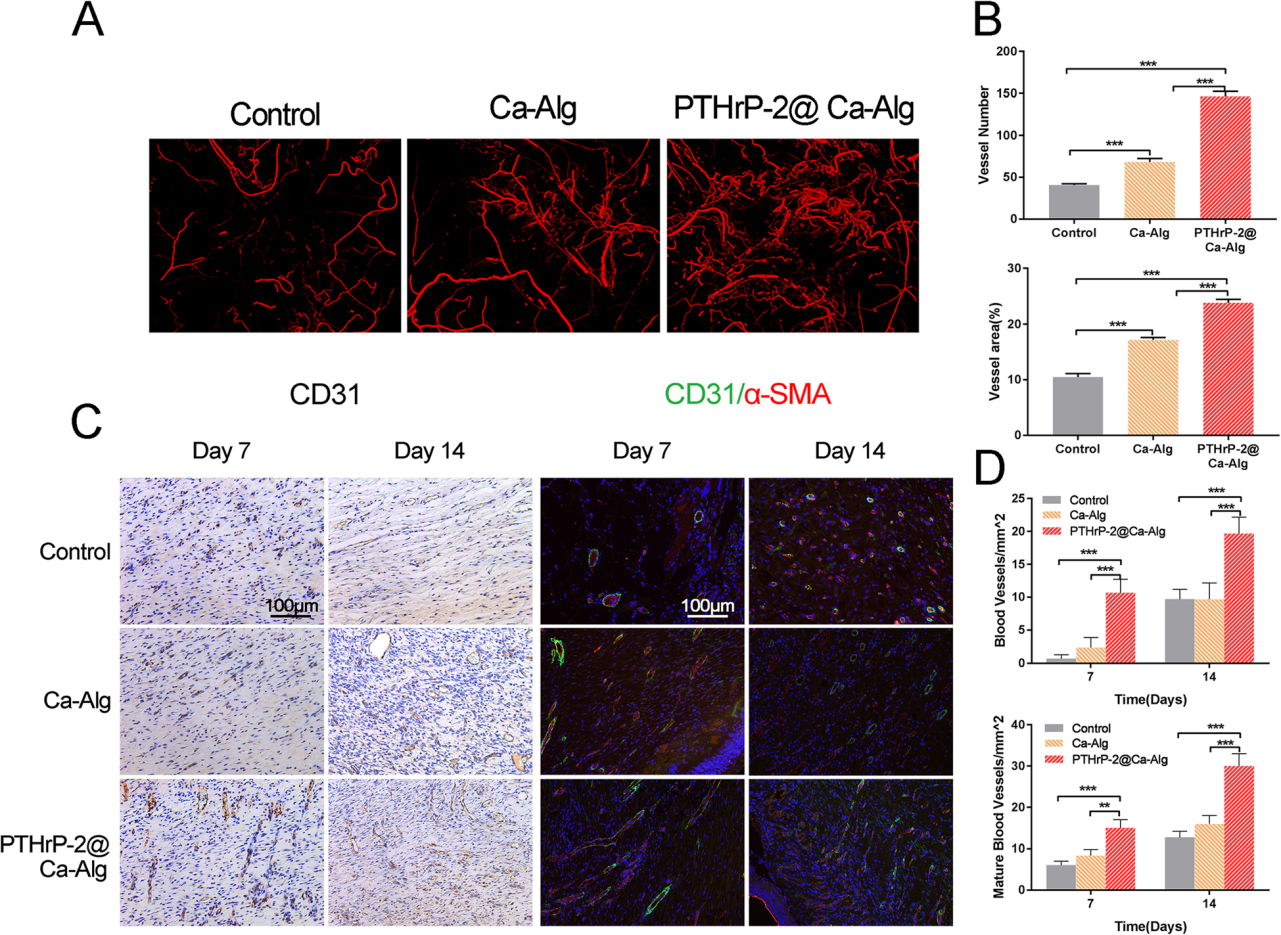
**a** 3D reconstructed images showing the new blood vessels; **b** morphometric analysis of the new blood vessel area and the number of blood vessels. **c** IHC (Immunohistochemical staining for CD31) and IF (Immunohistochemical staining for CD31+ α-SMA) analysis of neovascularization in wound sites of control, Ca-Alg, PTHrP-2@Ca-Alg groups. The red arrow represents the neovascularization (oval or circular shape). Smooth muscle cells (α-SMA) and EC (CD31) were stained with green, red and blue nuclei. Green and red co-staining represents mature blood vessels. **d** Quantitative analysis of neovascularization and mature blood vessels at Day 7 and Day 14 post-operation. Micro-CT evaluation of blood vessel formation in diabetic rat treated with different treatment therapies at Day 14 post-surgery

Fourteen days after surgery, Micro CT was used to evaluate the vascular formation of Ca-Alg-treated, PTHrP-2@Ca-Alg-treated and untreated wounds. The reconstructed three-dimensional images (Fig. 2a) showed that the vascular density in the PTHrP-2@Ca-Alg-treated group was significantly higher than that in the other two groups. The quantitative data analysis of the number of newly formed blood vessels (Fig. 2b) and the area and number of blood vessels showed that these variables were significantly higher in the PTHrP-2 treatment group than in the Ca-Alg group and control group.

CD31 immunohistochemical staining and CD31/α-SMA dual immunofluorescence staining of the wound tissue at 7 and 14 days after surgery showed new blood vessel formation and mature blood vessels (Fig. 2c). The quantitative data analysis of the newly formed vascular density, i.e., the number of CD31-positive cells per mm^2^, confirmed the increase in the number of wound vessels following treatment with PTHrP-2@Ca-Alg (Fig. 2d). At day 14, blood vessel density was much higher in the PTHrP-2@Ca-Alg group than in other two groups. The number of mature blood vessels increased in all three groups from the 7th day to the 14th day after the wound surface operation but was lower than the number of new blood vessels in each group. The number of mature blood vessels on the wound surface significantly increased in the PTHrP-2@Ca-Alg group relative to the numbers in the other groups on the 14th day after surgery.

**Fig. 3:**
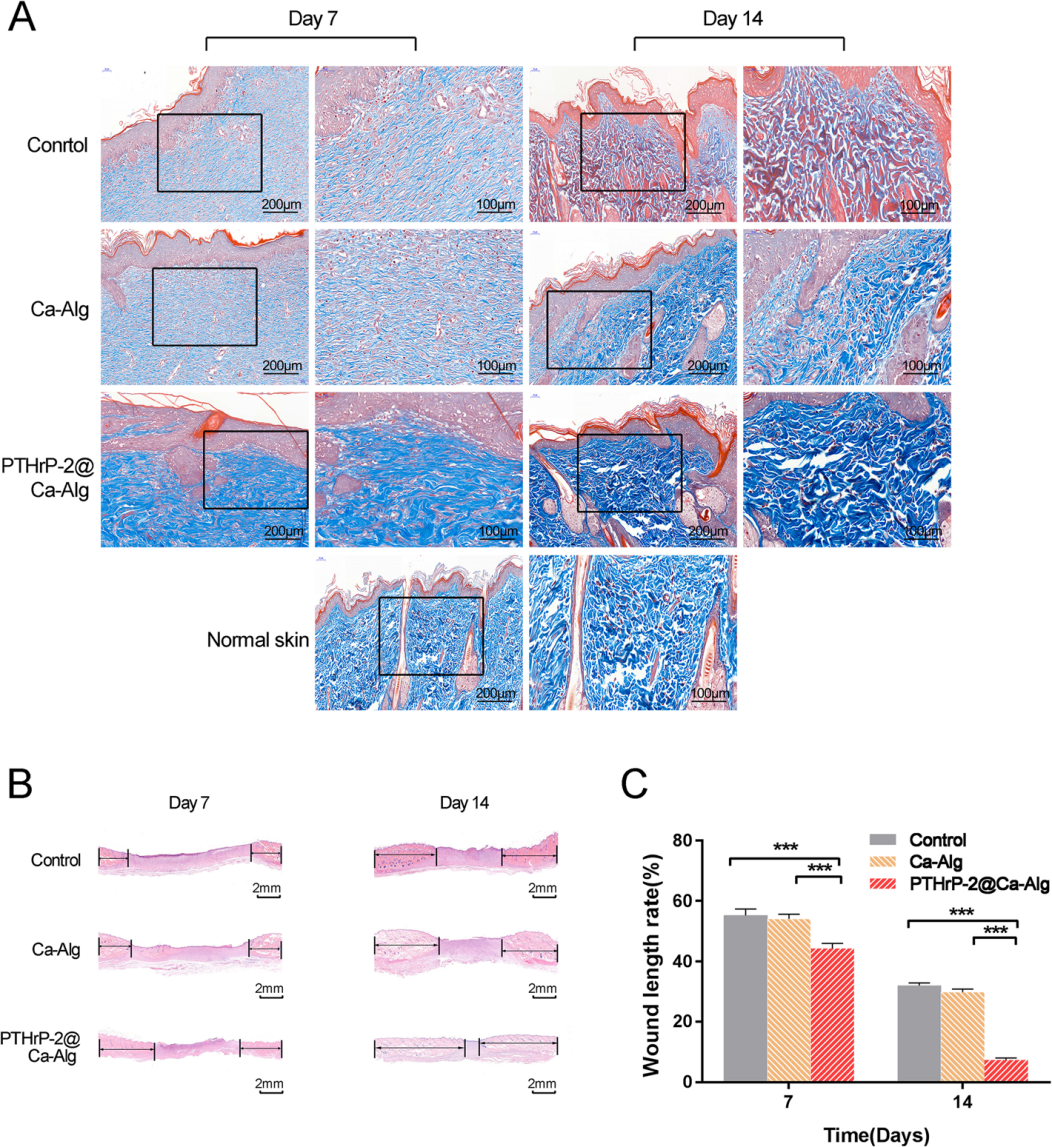
**a** Four different treatments were used for the sections stained with Masson’s trichrome at 7 and 14 days after surgery, showing collagen deposition. **b** Images of H&E stained wound sections of the defects treated with different treatment therapies at 7 and 14 days after surgery. The black arrows indicate the neoepithelium. **c** Statistical analysis of the total length of neonatal epithelium at 7 and 14 days

According to the histological analysis of the Masson’s trichrome staining, there was a significant difference in treatment effect among the three groups (Fig. 3a). Compared with the control group, the PTHrP-2@Ca-Alg-treated group showed more extensive collagen deposition and greater collagen fiber thickness. The processed images of the PTHrP-2@Ca-Alg-treated group revealed improved arrangement of collagen fibers, similar to that of normal skin, which reflected the positive roles of PTHrP-2 in ECM deposition and collagen alignment. In general, the PTHrP-2@Ca-Alg group had greater numbers structures resembling hair follicles and sebaceous glands than did the other groups. According to the optical microscopy of H&E staining (Fig. 3b), new epithelial tissue formed in the wounds of the three groups. The initial width of each wound was 2 cm; in the figure. The black arrow indicates the length of the new epithelium. As shown in the figure (Fig. 3c), the wound healing effect in the PTHrP-2@Ca-Alg treatment group was significantly better than that in the other two groups on days 7 and 14.

### In vitro study of PTHrP-2 effects

**Fig. 4:**
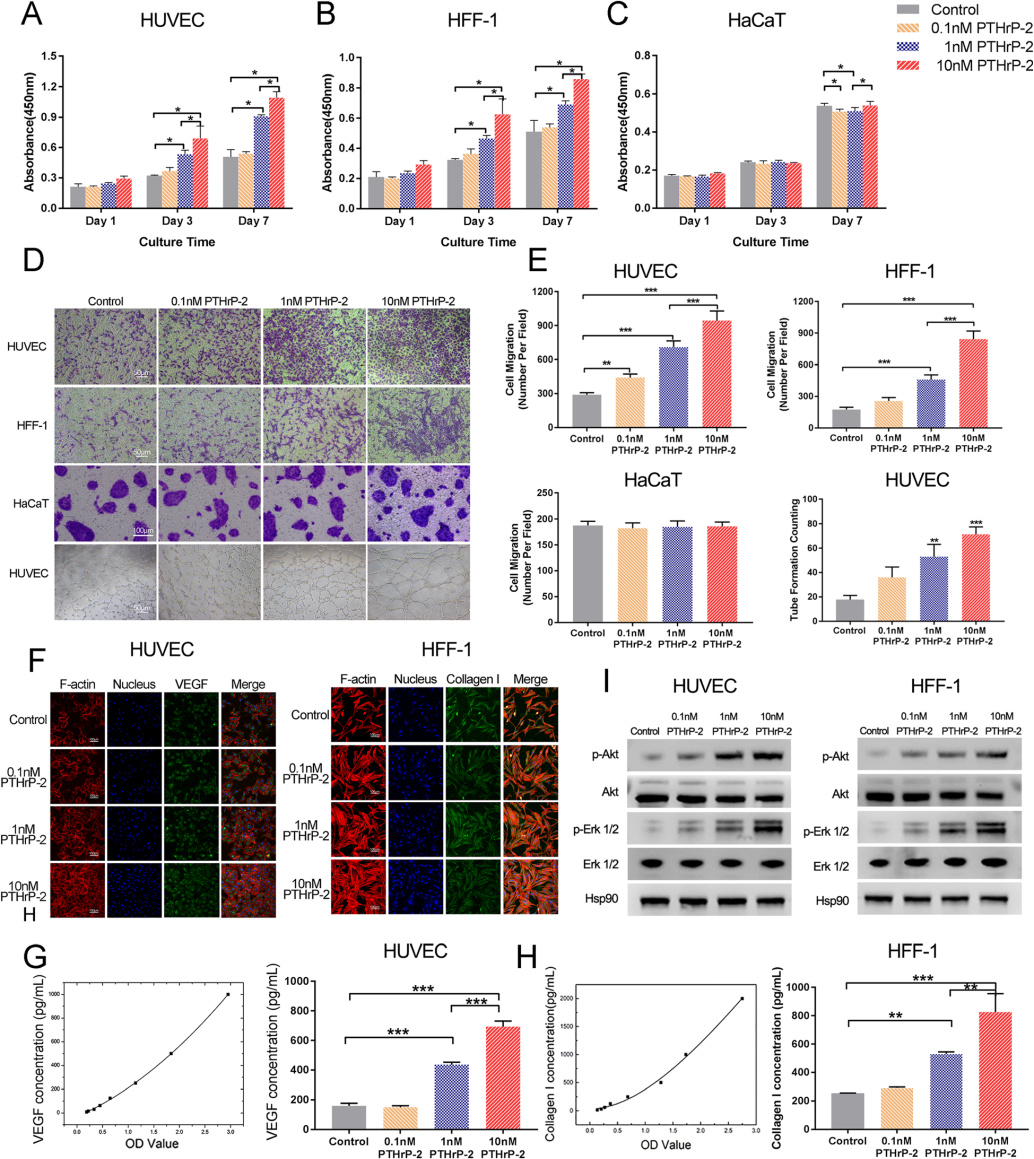
Proliferation of HUVECs (**a**), HFF-1 cells (**b**), HaCaTs (**c**) incubated for 0, 1, 3, or 7 days in conditioned medium with different drug concentrations from days 0 and 6. **d** Effects of PTHrP-2 on migration of HUVECs, HFF-1 cells and HaCaTs and the tube formation assay of HUVECs. **e** Quantitation of HUVECs, HFF-1 cells and HaCaTs migration (violet stained cells) using a Transwell chamber. The quantitative evaluation of the number of nodes formed in the culture plate with different drug concentrations after 8 h. **f** Immunofluorescence images of HUVECs and HFF-1 incubated in each group on day 3. Cytoskeleton and cell nuclei are stained red and blue, VEGF and Collagen I are stained green in the picture taken by The laser scanning confocal microscopy. **g** VEGF and Collagen I secretion by HUVEC and HFF-1 incubated for 3 days in media with different drug concentrations. **h** Akt and Erk1/2 phosphorylation level in HUVEC and HFF-1 treated with different drug concentrations

HUVECs (Fig. 4a), HFF-1 cells (Fig. 4b) and HaCaTs (Fig. 4c) were cultured in medium containing different concentrations for 1, 3 and 7 days, respectively, and the proliferation of these cell lines was shown in Fig. 2. In HUVECs and HFF-1 cells, the proliferation rates of cells in the treatment groups were higher than that of cells in the untreated groups, and the proliferation effect became more pronounced as the concentration increased beginning at 3 days. At 7 days, we observed that PTHrP-2 stimulated the proliferation of HUVECs and HFF-1 cells; 10 nM PTHrP-2 had the strongest effect on cell proliferation. However, in the CCK-8 assay, we found that PTHrP-2 had no significant effect on proliferation of HaCaTs, indicating that there was no cytotoxicity.

The transwell assays showed that the numbers of migrated cells in the treatment groups were significantly higher than those in the untreated groups. Among the four groups, the group treated with 10 nM PTHrP-2 exhibited the strongest migration capacity. By measuring the tube formation activity of HUVECs, we determined the angiogenic potential of PTHrP-2 at different concentrations. The HUVECs treated with PTHrP-2 formed elongated tube structures on the substrate gel substrate layer after 8 h of incubation, whereas without PTHrP-2 treatment, the HUVECs formed incomplete or sparse tubular networks. The HUVECs treated with 10 nM PTHrP-2 produced the most blood vessels among the treatment groups, and the blood vessels were largely complete (Fig. 4d, e).

In Fig. 4f, the cytoskeleton was stained red by phalloidin, the nucleus was stained blue by DAPI, and the VEGF secreted by the cells was green. At 3 days, the amounts of blue and green fluorescence in the treatment groups were significantly greater than those in the untreated group. PTHrP-2 strongly stimulated VEGF secretion from HUVECs and HFF-1 cells. Among the four treatments, 10 nM PTHrP-2 was the most effective. To further explore the mechanism of the PTHrP-2 effect on HUVEC and HFF-1 cells, the levels of VEGF secreted from cells cultured in different concentrations of PTHrP-2 were detected by an ELISA kit. In the PTHrP-2 treatment group, the VEGF content in the supernatant of cultured HUVECs was the highest after 3 days, and 10 nM PTHrP-2 provided the best conditions for HUVEC secretion of VEGF. Similar results were obtained in HFF-1 cells. After treatment in the 10 nM PTHrP-2 group, type I collagen of HFF-1 cells was the highest, followed by 1 nM PTHrP-2. However, in the HFF-1 cells, there was no difference in type I collagen levels between the 0.1 nM and untreated treatment (Fig. 4g).

To explore the mechanism of PTHrP-2 action at the protein level, Western blotting was performed in the untreated groups and the groups treated with different concentrations of PTHrP-2. The results (Fig. 4h) suggest that PTHrP-2 may activate the PI3K/Akt and Erk1/2 signaling pathways. Compared with the untreated group, the treatment groups showed increased phosphorylation of Akt and Erk, which indicated the activation of the two signaling pathways in HUVECs and HFF-1 cells. Among different groups, the 10 nM treatment group exhibited the most pronounced effect.

### Evaluation of the PTHrP-2-treated exosomes

#### Characterization of PTHrP-2-treated exosomes

**Fig. 5:**
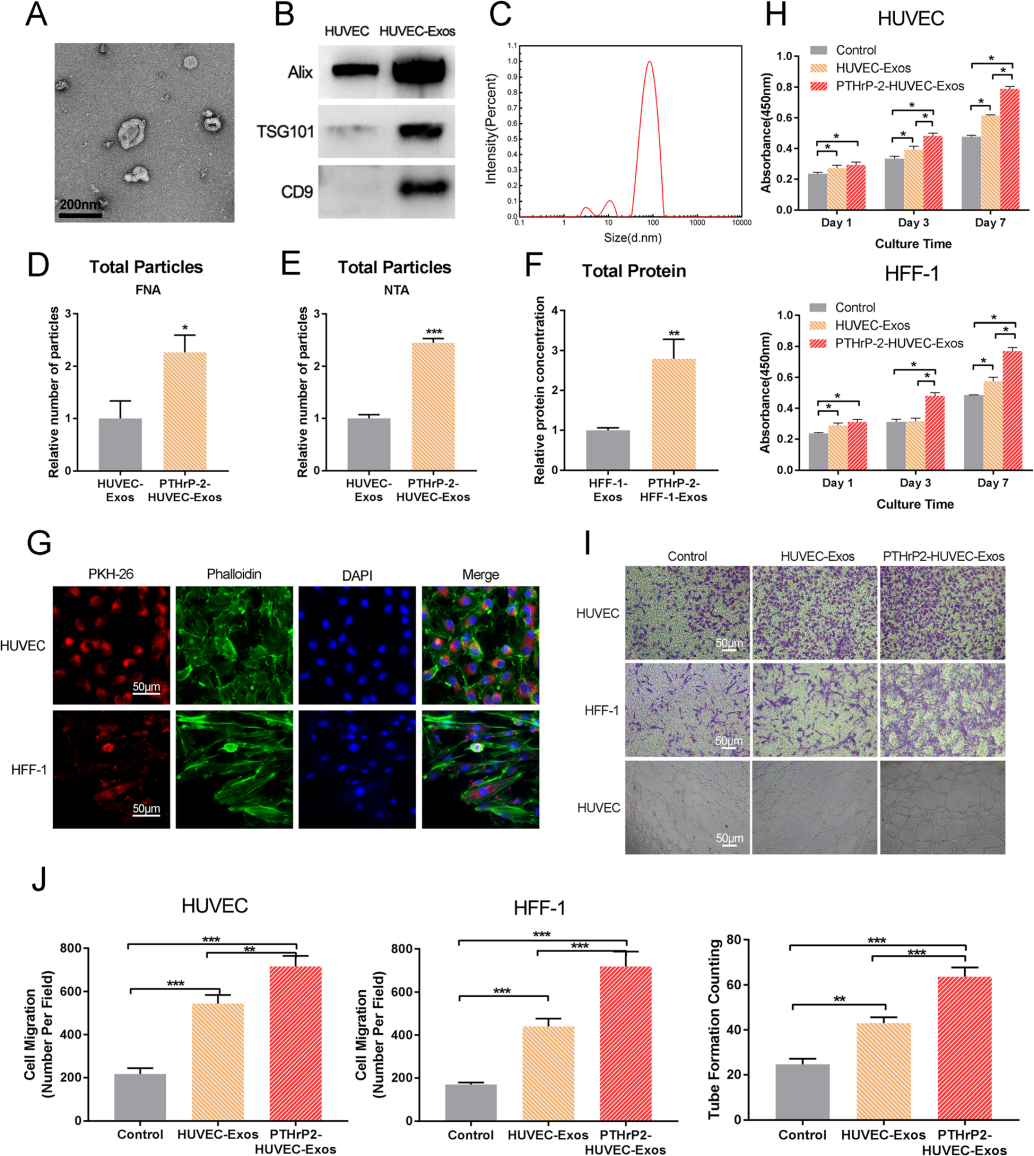
**a** TEM images of HUVEC-Exos. **b** Exosome surface markers detected by Western blotting (Alix, Tsg101, CD9). The experiment was repeated three times in order to confirm the stability of the phenomena. **c** Size distribution of exosomes. Particle concentration, particle size and video frame of exosomes were analyzed by FNA (**d**) and NTA (**e**). Total protein levels (**f**) in HUVEC-Exos and PTHrP-2- HUVEC-Exos. (**g**) The uptake of exosomes by HUVECs and HFF-1 cells. Cytoskeleton, exosomes and cell nuclei are stained green, red and blue in the picture taken by the laser scanning confocal microscopy. **h** Proliferation of HUVEC and HFF-1 incubated for 0, 1, 3, or 7 days in conditioned medium with HUVEC-Exos and PTHrP-2- HUVEC-Exos from days 0 and 6. **i** Effects of HUVEC-Exos on migration of HUVECs, HFF-1 cells and the tube formation assay of HUVECs. **j** Quantitation of HUVECs and HFF-1 cells, migration (violet stained cells) using a Transwell chamber. The quantitative evaluation of the number of nodes formed in the culture plate with different conditions of culture after 8 h

**Fig. 6:**
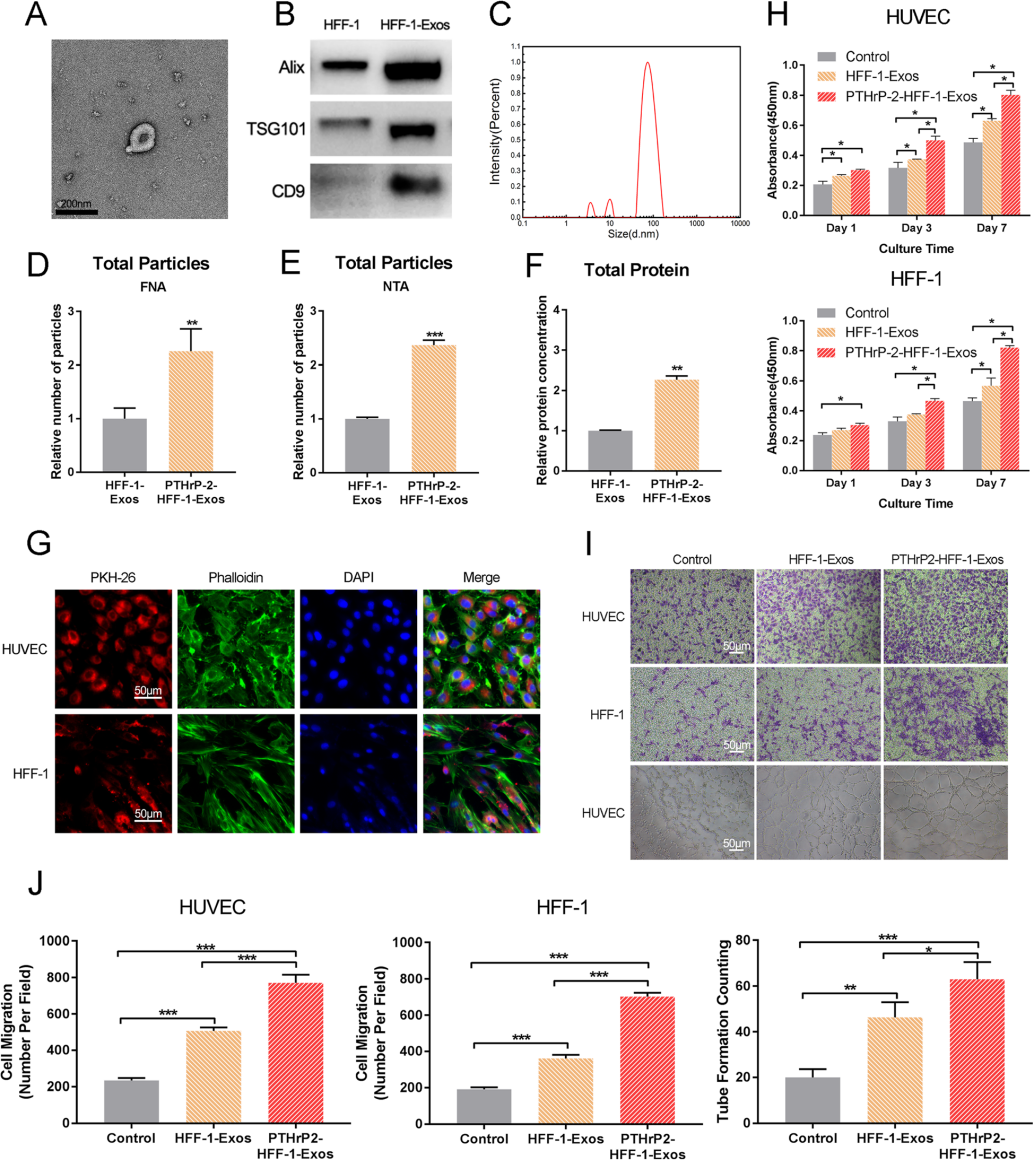
**a** TEM images of HFF-1-Exos. **b** Exosome surface markers detected by Western blotting (Alix, Tsg101, CD9). The experiment was repeated three times in order to confirm the stability of the phenomena. **c** Size distribution of exosomes. Particle concentration, particle size and video frame of exosomes were analyzed by FNA (**d**) and NTA (**e**). Total protein levels (**f**) in HFF-1-Exos and PTHrP-2-HFF-1-Exos. **g** The uptake of exosomes by HUVECs and HFF-1 cells. Cytoskeleton, exosomes and cell nuclei are stained green, red and blue in the picture taken by the laser scanning confocal microscopy. **h** Proliferation of HUVECs and HFF-1 cells incubated for 0, 1, 3, or 7 days in conditioned medium with HFF-1-Exos and PTHrP-2-HFF-1-Exos from days 0 and 6. **i** Effects of HFF-1-Exos on migration of HUVECs and HFF-1 cells and the tube formation assay of HUVECs. **j** Quantitation of HUVECs and HFF-1 cells migration (violet stained cells) using a Transwell chamber. The quantitative evaluation of the number of nodes formed in the culture plate with different conditions of culture after 8 h

The nanoparticles purified from HUVECs and HFF-1 cells treated with PTHrP-2 were characterized by TEM, DLS and Western blotting. TEM (Fig. 5a and 6a) experiments with PTHrP-2-treated exosomes showed that most of the extracted nanoparticles were spherical or cup-shaped, which indicated the presence of exosomes. We observed the presence of exosome markers, such as Alix, TSG101 and CD9, by Western blotting (Fig. 5b and 6b). These markers confirmed that the particles were exosomes. The sizes of the PTHrP-2-treated exosomes were directly determined using a DLS system called the Nanosizer system, which ranged from 40 to 100 nm (Fig. 5c and 6c) When the samples were further concentrated for data analysis, we found that the exosome concentration in the treated group was approximately 1.5 times greater than that in the untreated group through FNA (Fig. 3 and 4d) and NTA (Fig. 5e and 6e) detection. After exosome proteins were extracted, BCA protein assay (Beyotime, China) was performed, and the data were in accordance with the concentration ratio (Fig. 5f and 6f). The cytoskeleton, PKH26-labeled exosomes (PKH26-Exos) and cell nuclei were stained green, red and blue, respectively, in the images (Fig. 5g and 6g) collected with laser scanning confocal microscopy. PKH26-Exos were located in the area around the nucleus. A red area in the perinuclear region was observed in more than 90% of the HUVECs and HFF-1 cells. These data indicated that the exosomes could be successfully internalized by HUVECs and HFF-1 cells.

#### In vitro study of the PTHrP-2-treated exosomes

To investigate the effect of PTHrP-2 on exosomes, we extracted exosomes from untreated and treated HUVECs and HFF-1 cells. The extracted exosomes were tested for their effects on the migration of HUVECs and HFF-1 cells as described above. Relative to control treatment and treatment with the untreated exosomes, treatment with the PTHrP-2-treated exosomes significantly increased the proliferation (Fig. 5h and 6h) and migration of HUVECs and HFF-1 cells. Relative to the untreated group, the PTHrP-2-HUVEC-Exos group exhibited significantly increased proliferation and migration capacity of HaCaTs. Corresponding increases were not observed in the PTHrP-2-HFF-1-Exos group. Similarly, in the tube formation experiments with HUVECs, the greatest number of nodes formed in the group treated with the PTHrP-2-treated exosomes. These data indicated that the group treated with PTHrP-2 treated exosomes was superior to the other two groups in the promotion of angiogenesis (Fig. 5i, j and 6i, j). All comparisons were based on the same total protein level.

#### The effect of PTHrP-2-treated-Exos on HaCaTs

**Fig. 7:**
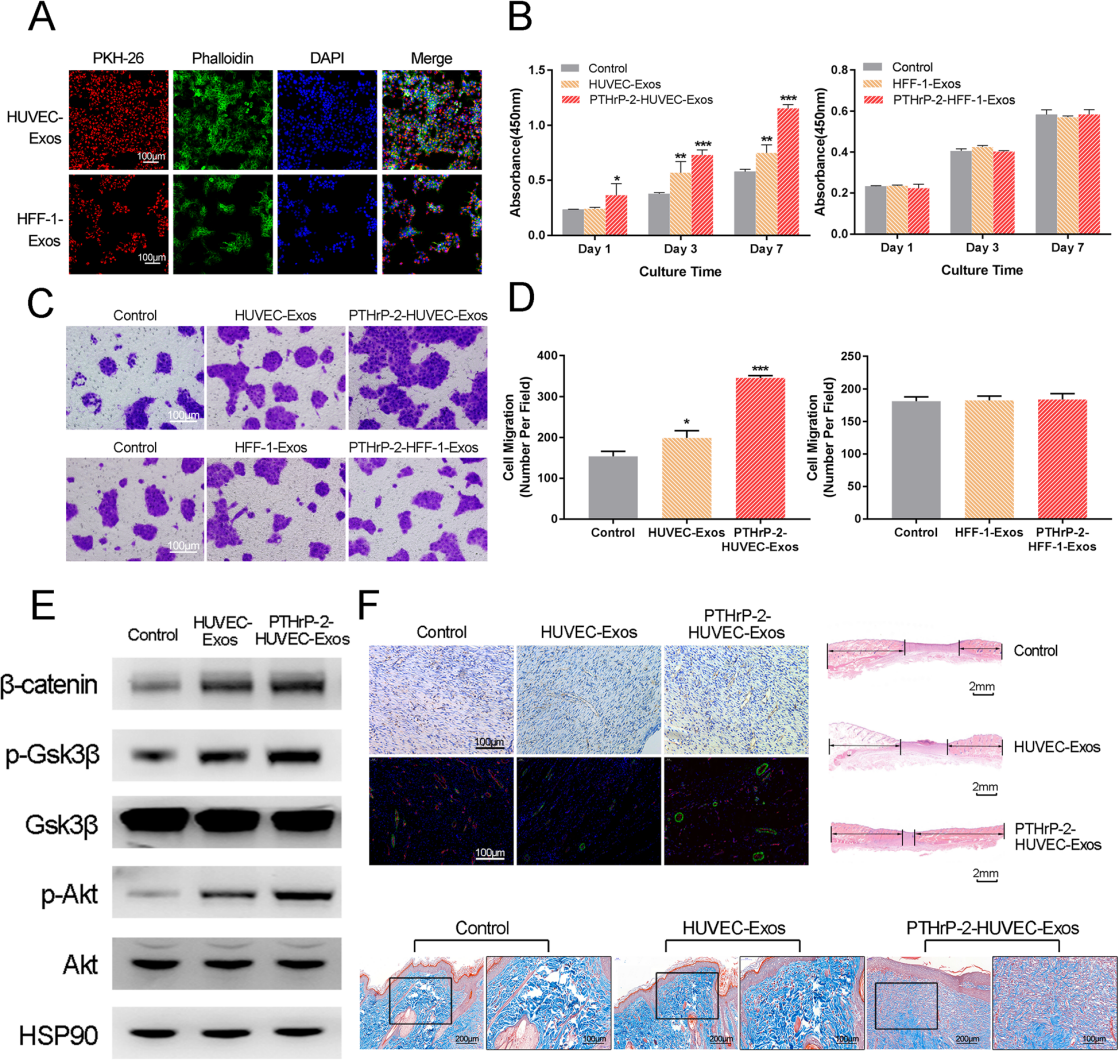
**a** The uptake of exosomes by HaCaTs (**b**) Proliferation of HaCaTs incubated for 0, 1, 3, or 7 days in conditioned medium with PTHrP-2-HUVEC-Exos and PTHrP-2-HFF-1-Exos from days 0 and 6. **c** The migration of HaCaTs conditioned medium with PTHrP-2-HUVEC-Exos and PTHrP-2-HFF-1-Exos. **d** Protein phosphorylation levels of Gsk3β and AKT analyzed by Western blot (**e**) In vitro results of PTHrP-2-HUVEC-Exos

As shown in Fig. 7a, after staining with PKH26, HUVEC-Exos and HFF − 1-Exos were clustered near the nucleus of HaCaTs. This result indicated that exosomes could phagocytize HaCaTs and played a certain role. According to the results of the CCK-8 assay (Fig. 7b), HUVEC-Exos promoted HaCaTs, and PTHrP-2-HUVEC-Exos significantly enhanced this effect. No marked proliferation effects of HFF-1-Exos and PTHrP-2-HFF-1-Exos on HaCaTs were observed, and there was no significant difference between the groups. In the verification of HaCaTs migration experiment (Fig. 7c), the enhancement of PTHrP-2-HUVEC-Exos on HaCaTs migration ability was confirmed. Similarly, HFF-1-Exos and PTHrP-2-HFF-1-Exos had no significant effects on HaCaTs. According to the characterization results of HaCaTs, Western blot experiments (Fig. 7d) were conducted on HaCaTs co-cultured with HUVEC-Exos and PTHrP-2-HUVEC-Exos. The classical pathway of HaCaTs was selected for verification. It could be seen from the figure that PTHrP-2-HUVEC-Exos activated the PI3K/AKT signaling pathway and upregulates the expression of β-catenin by promoting the phosphorylation of Gsk3β.

#### The effect of PTHrP-2-HUVEC-Exos in vivo

Based on the results of the in vitro experiments, HUVEC-Exos and PTHrP-2-HUVEC-Exos were used for the treatment of diabetic rat wounds by subcutaneous injection. In the HE staining, Masson staining, CD31 immunohistochemical staining and CD31/α-SMA dual immunofluorescence staining experiments, PTHrP-2-HUVEC-Exos group was found to yield significantly better effects than the other two treatments. After 14 days of treatment with PTHrP-2-HUVEC-Exos, the rates of epithelialization, angiogenesis and collagen arrangement of the wound surface were significantly enhanced (Fig. 7e).

## Discussion

Skin tissue is composed of a dermal layer and corium layer and three cell types: endothelial cells, fibroblasts and epithelial cells. Wound healing can be divided into four phases: a coagulation phase, an inflammation phase, a reconstruction phase and a maturation phase [30]. All three cell types are involved in these phases. Diabetic conditions impair the activation of these three cell types, impairing wound healing and leading to wounds [31]. In healing wounds, the three cell types are equally important. Hyperglycemia caused by diabetes can destroy the microvascular structure, thereby affecting the functions of blood vessels and the ability of cells to deliver oxygen [32, 33]. Collagen, a component of the corium layer, can also be impaired by hyperglycemia [34]. Furthermore, some studies have reported that hyperglycemia can inhibit epithelium cell proliferation and migration. These phenomena may inhibit wound healing and lead to the formation of wounds [35]. The three types support and protect each other. Simultaneous activation of the three cell types have no simple additive effects but rather a synergistic effect. Different from traditional bioactive factors, PTH has multiple roles in wound healing via multicellular activation. In vivo, PTHrP-2 induced rapid wound closure. H&E staining revealed increased re-epithelization of the wound by PTHrP-2. Masson trichrome staining revealed denser collagen deposition and a better collagen array, approaching that of normal skin, due to PTHrP-2. Immunofluorescence and immunohistochemistry for CD31 and a-SMA and microfil perfusion revealed a denser neovascularization and more mature capillary structure under PTHrP-2 treatment. These results suggested that PTHrP-2 was effective in promoting wound healing and that its healing effects were multicellular. PTHrP-2 was a multifunctional factor that exerts synergistic effects on the three cell types and wound healing.

To clarify the mechanism of action of PTHrP-2 in wound healing, we conducted in vitro experiments to study the effects of PTHrP-2 on the three cell types. Endothelial cells and fibroblasts exhibited activation under PTHrP-2 conditions. Proliferation, migration, capillary-structure formation, VEGF and type I collagen secretion were promoted by PTHrP-2. In previous studies, PTH-treated cells were found to play major roles via PTH1R and the downstream Akt/Erk 1/2 pathway [36, 37]. Furthermore, the Akt/Erk 1/2 pathway is important in angiogenesis and fibrogenesis. Therefore, we examined Akt/Erk 1/2 pathway change under PTHrP-2 conditions in endothelial cell and fibroblasts. The results indicated that the Akt/Erk 1/2 pathway was a major pathway through which PTHrP-2 acts on endothelial cells and fibroblasts.

However, these promoting effects were not seen in HaCaTs under PTHrP-2 intervention. Proliferation and migration of HaCaTs were not enhanced by PTHrP-2. The in vitro results were not in line with the in vivo results. HaCaTs do have PTH receptors on their surfaces. Some studies reported that PTH promotes antimicrobial peptide expression on epithelial cells [38]. Our experiment results and previous studies indicated that PTHrP-2 might have major antibacterial effects on epithelial cells but not proliferation or migration activity. We formulated a second hypothesis to explain the promotion of re-epithelization in the in vivo experiment. We hypothesized that PTHrP-2 promotes re-epithelization by altering the intercellular communication among the three cell types. The exosome, a newly discovered extracellular vesicle, was postulated as major mediator of intercellular communication in this study.

Exosomes from endothelial cells and fibroblasts without PTHrP-2 interference were carefully isolated, identified and added to the three cell types. Exosomes from endothelial cells and fibroblasts without PTHrP-2 interference had some self-activation and interactivation abilities. In addition, endothelial cell exosomes had some ability to promote HaCaTs proliferation and migration. These results suggested exosomal-mediated cellular interactions in skin tissue. Endothelial cells, fibroblasts and epithelial cells underwent natural exosomal interactions to maintain skin tissue homeostasis.

Exosomes from endothelial cells and fibroblasts under PTHrP-2 interference showed stronger self-activation and interactivation abilities than did those without such interference. Importantly, PTHrP-2-stimulated endothelial cell exosomes exhibited significant promotion effects on HaCaTs proliferation and migration, which might explain PTHrP-2’s ability to cause re-epithelization. Previous studies reported that certain drugs and environments, such as hypoxia, might alter exosome contents and thereby alter the intercellular messages communicated by exosomes [39–41]. The exosome counts revealed that PTHrP-2 enhanced exosome secretion. Through enhancing exosome production and strengthening exosome abilities, PTHrP-2 reinforced the intercellular interactions among endothelial cells, fibroblasts and epithelial cells and produced an exosomal intercellular network.

When investigating the mechanism of action of exosomes obtained following PTHrP-2 treatment, we selected the PI3K-Akt pathway for HaCaTs due to its canonical role in re-epithelization [42, 43]. Gsk3β is a typical regulation of the negative Wnt signaling pathway. Many researchers have described PI3K/AKT and Gsk3β/β-catenin signals as playing a key role in skin development and wound healing [44]. Based on the findings of previous studies, Gsk3β/β-catenin and PI3K/Akt signaling pathway was selected for verification. The results showed that canonical pathways were activated by PTHrP-2-interfered exosomes. This finding provided a good explanation for the enhanced proliferation and migration ability of HaCaTs due to PTHrP-2-HUVEC-Exos. We did not identify the factor that initiated these canonical pathways; this topic will be explored in our next study.

Tests of the effects of PTHrP-2-interfered exosomes on wound healing in vivo were conducted. Both natural endothelial cell exosomes and PTHrP-2-interfered exosomes exhibited wound healing properties. However, PTHrP-2-interfered exosomes exhibited much stronger healing abilities than natural endothelial cell exosomes in proangiogenesis, profibrogenesis and re-epithelization. The results confirmed our speculation.

In this study, we carried out in vivo and in vitro experiments from the perspective of drugs to promote tissue repair. Although the results of this study were satisfactory, there were still some defects in revealing the underlying mechanism of wound in diabetes mellitus and the factors that led to lead PTHrP-2-stimulated enhancement of exosome abilities. In the follow-up study, we consider to dig deeply into the pathogenesis of diabetes. Further studies will be conducted using in vivo and in vitro models of diabetes, such as high-sugar models or transgenic animals and the factors and related pathways will be investigated in our future work.

## Conclusion

In this study, we hypothesized that a PTH derivative could be applied to wound healing for its multicellular stimulatory effects. Our first in vivo study validated our hypothesis, but the in vitro results did not provide evidence of the promotion of wound re-epithelization by PTHrP-2. Therefore, we further hypothesized that PTHrP-2 altered intercellular communication among endothelial cells, fibroblasts and epithelial cells. Exosomes were isolated from PTHrP-2 stimulated endothelial cells and showed a strong ability to activate epithelial cells. The findings indicated that the mechanism of PTHrP-2 in promoting wound healing could be attributed to direct stimulation and indirect exosomal activity. PTHrP-2 was an ideal multifunctional wound healing agent that has multicellular synergistic effects.

## Supplementary information

**Additional file 1 Fig. S1** Biocompatibility of Ca-Alg in HUVECs (A), HFF-1 cells (B) and HaCaTs (C). The release curve of PTHrP-2@Ca-Alg (D).

## Data Availability

The datasets generated during and/or analyzed during the current study are available from the corresponding author upon reasonable request.

## Abbreviations

b-FGF: basic fibroblast growth factor
BSA: Bovine serum albumin
Ca-Alg: Calcium alginate hydrogel
Col I: Collagen I
ECGS: Endothelial Cell Growth Supplement
ECL: Enhanced chemiluminescence
EGF: Epithelial cell growth factor
ELISA: Enzyme-linked immunosorbent assay
FNA: Flow NanoAnalyzer
Glu: Glutamic acid
HaCaT: HaCaT immortalized human epidermal cells
HUVEC: Human Umbilical Vein Endothelial Cells
NTA: Nanoparticle tracking analysis
PBS: Phosphate Buffered Saline
PTH: Parathyroid hormone
PTHrP-2: Parathyroid hormone reletaed peptide
SDS-PAGE: Sodium dodecyl sulfate-polyacrylamide gel electrophoresis
Ser: Serine
VEGF: Vascular Endothelial Grown Factor
