# Correction to: PTH Derivative promotes wound healing via synergistic multicellular stimulating and exosomal activities

**DOI:** 10.1186/s12964-020-00593-y

**Published:** 2020-05-19

**Authors:** Yi-Fan Shen, Jing-Huan Huang, Kai-Yang Wang, Jin Zheng, Lin Cai, Hong Gao, Xiao-Lin Li, Jing-Feng Li

**Affiliations:** 1 Department of Orthopaedic Surgery, Shanghai Jiao Tong University Affiliated Sixth People’s Hospital, Shanghai, People’s Republic of China; 2 Department of Orthopedics, Zhongnan Hospital of Wuhan University, Wuhan, People’s Republic of China; 3 Department of Neurology, Union Hospital, Tongji Medical College, Huazhong University of Science and Technology, Wuhan, People’s Republic of China

**Correction to: Cell Commun Signal (2020) 18:40**

**https://doi.org/10.1186/s12964-020-00541-w**

Following publication of the original article [1], two mistakes were noticed in Fig. 4 and Fig. 6. The pictures describing the effects of 0.1 nM PTHrP-2 group on migration of HUVEC in Fig. 4 and Control and HFF-1-Exos groups on migration of HFF-1 cells in Fig. 6 are incorrect. The correct figures are supplied below in this correction article. The figure legends were not changed.

**Fig. 4:**
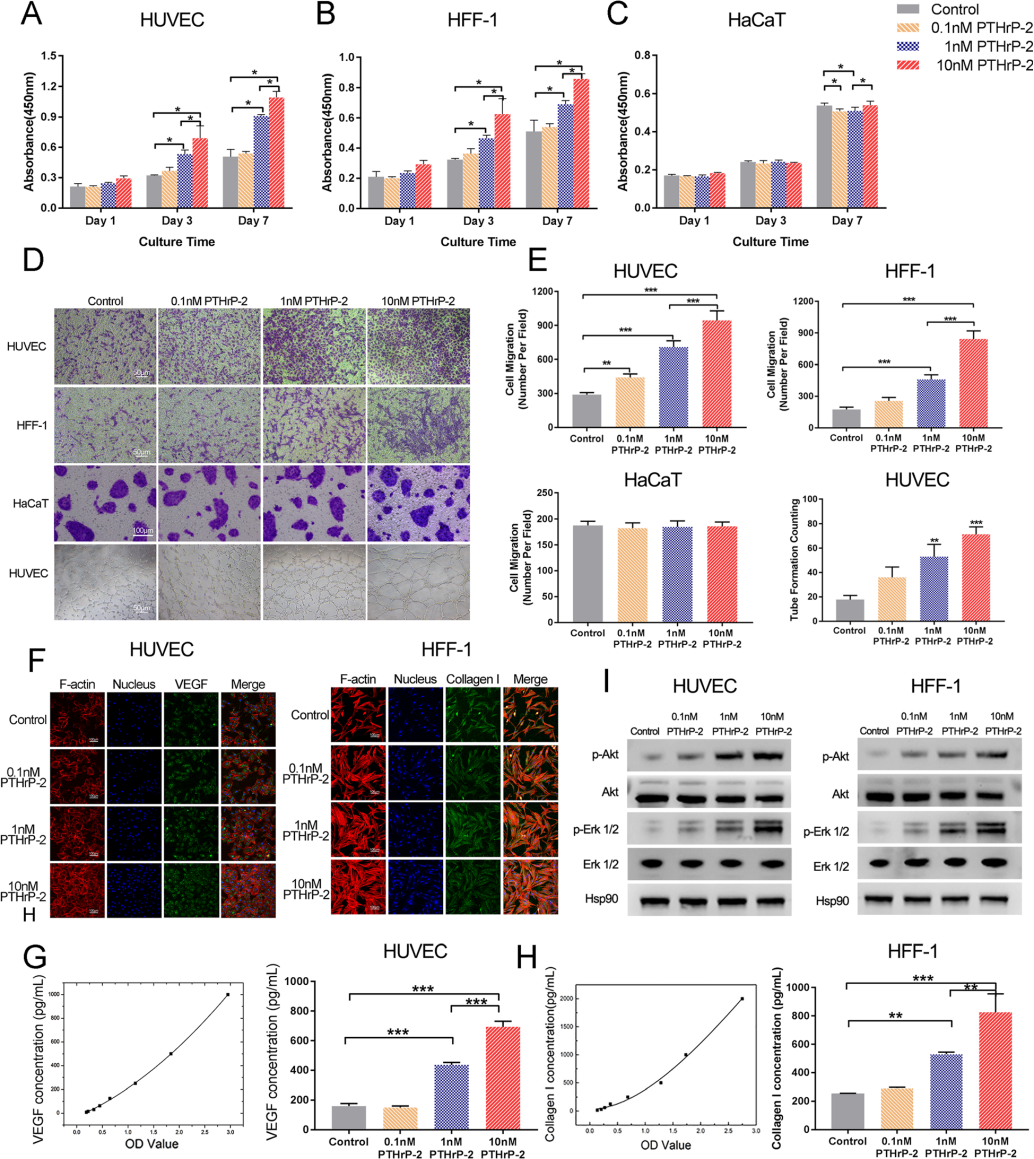
Proliferation of HUVECs (**a**), HFF-1 cells (**b**), HaCaTs (**c**) incubated for 0, 1, 3, or 7 days in conditioned medium with different drug concentrations from days 0 and 6. **d** Effects of PTHrP-2 on migration of HUVECs, HFF-1 cells and HaCaTs and the tube formation assay of HUVECs. **e** Quantitation of HUVECs, HFF-1 cells and HaCaTs migration (violet stained cells) using a Transwell chamber. The quantitative evaluation of the number of nodes formed in the culture plate with different drug concentrations after 8 h. **f** Immunofluorescence images of HUVECs and HFF-1 incubated in each group on day 3. Cytoskeleton and cell nuclei are stained red and blue, VEGF and Collagen I are stained green in the picture taken by the laser scanning confocal microscopy. **g** VEGF and Collagen I secretion by HUVEC and HFF-1 incubated for 3 days in media with different drug concentrations. **h** Akt and Erk1/2 phosphorylation level in HUVEC and HFF-1 treated with different drug concentrations

**Fig. 6:**
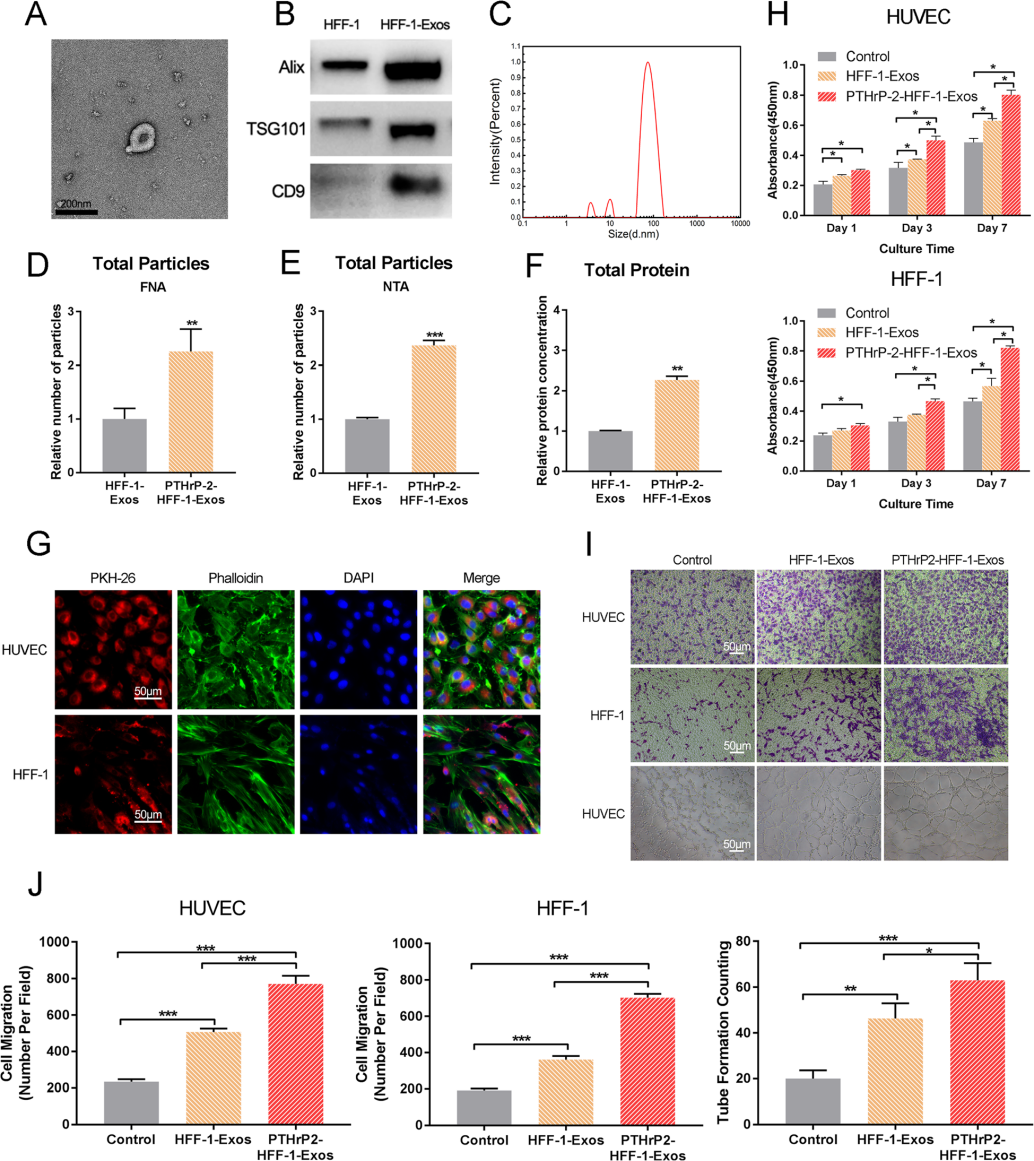
**a** TEM images of HFF-1-Exos. **b** Exosome surface markers detected by Western blotting (Alix, Tsg101, CD9). The experiment was repeated three times in order to confirm the stability of the phenomena. **c** Size distribution of exosomes. Particle concentration, particle size and video frame of exosomes were analyzed by FNA (**d**) and NTA (**e**). Total protein levels (**f**) in HFF-1-Exos and PTHrP-2-HFF-1-Exos. **g** The uptake of exosomes by HUVECs and HFF-1 cells. Cytoskeleton, exosomes and cell nuclei are stained green, red and blue in the picture taken by the laser scanning confocal microscopy. **h** Proliferation of HUVECs and HFF-1 cells incubated for 0, 1, 3, or 7 days in conditioned medium with HFF-1-Exos and PTHrP-2-HFF-1-Exos from days 0 and 6. **I** Effects of HFF-1-Exos on migration of HUVECs and HFF-1 cells and the tube formation assay of HUVECs. **j** Quantitation of HUVECs and HFF-1 cells migration (violet stained cells) using a Transwell chamber. The quantitative evaluation of the number of nodes formed in the culture plate with different conditions of culture after 8 h

The authors sincerely apologize for having this unintentional error in the article, and apologize for any inconvenience caused.

## References

[1] Shen Y, Huang J, Wang K (2020). PTH derivative promotes wound healing via synergistic multicellular stimulating and exosomal activities. Cell Commun Signal.

