# Supplementary figures and images for: PTH Derivative promotes wound healing via synergistic multicellular stimulating and exosomal activities
Source: Cell Commun Signal. 2020 Mar 9;18:40. doi: 10.1186/s12964-020-00541-w (PMC7063786; doi:10.1186/s12964-020-00541-w)

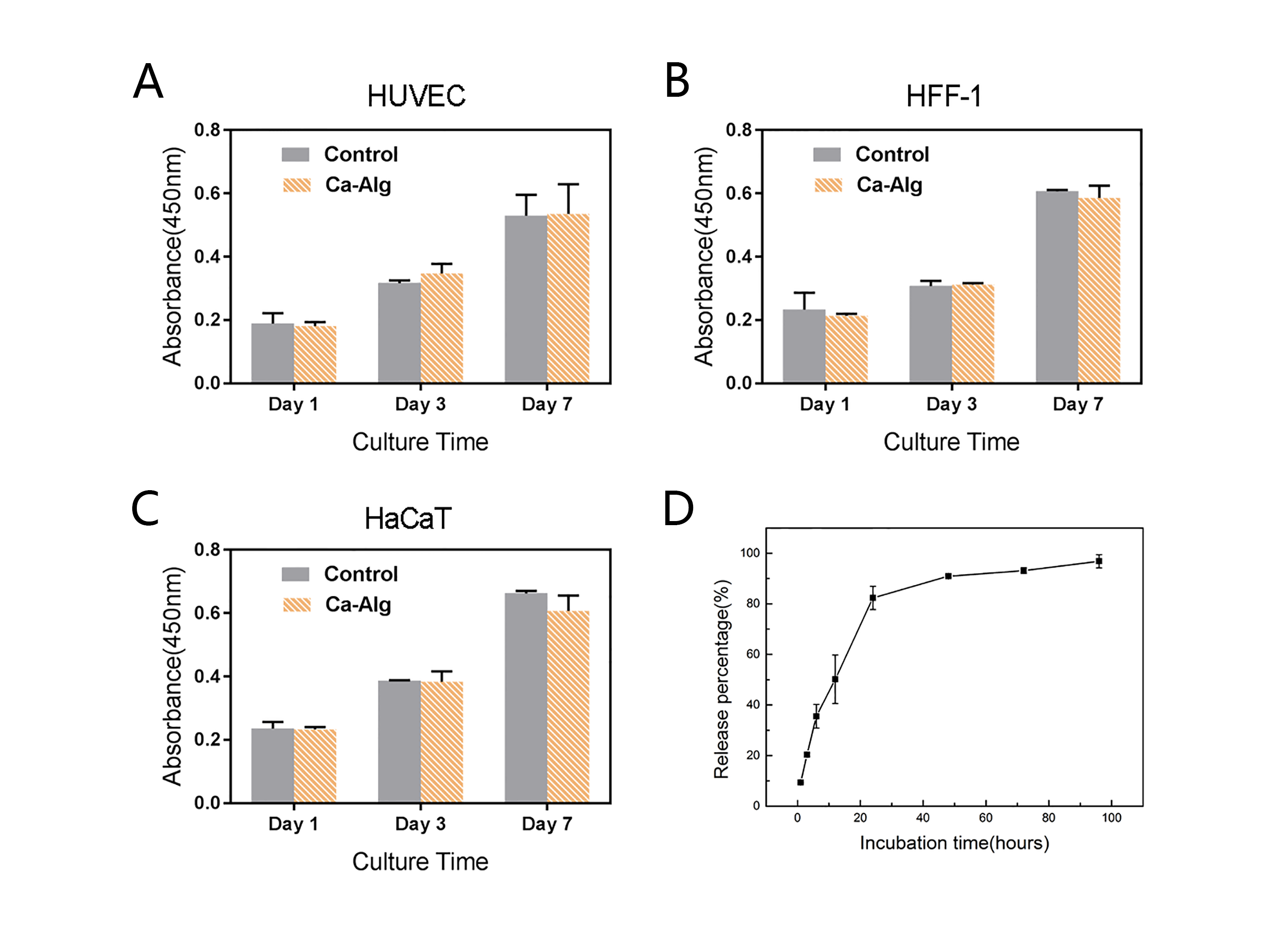

Supplement: Supplementary file 2 — Additional file 1 Fig. S1 Biocompatibility of Ca-Alg in HUVECs (A), HFF-1 cells (B) and HaCaTs (C). The release curve of PTHrP-2@Ca-Alg (D). [file 12964_2020_541_MOESM1_ESM.tif]
